# Estrogen receptor beta promotes lung cancer invasion via increasing CXCR4 expression

**DOI:** 10.1038/s41419-022-04514-4

**Published:** 2022-01-21

**Authors:** Shiqing Liu, Chengping Hu, Min Li, Jian An, Wolong Zhou, Jia Guo, Yao Xiao

**Affiliations:** 1 Department of Respiratory Medicine, Xiangya Hospital, Central South University, Changsha, 410008 China; 2 Xiangya Lung Cancer Center, Xiangya Hospital, Central South University, Changsha, 410008 China; 3 National Clinical Research Center for Geriatric Disorders, Xiangya Hospital, Central South University, Changsha, 410008 China; 4 Department of Thoracic Surgery, Xiangya Hospital, Central South University, Changsha, 410008 China; 5 Health Management Centre, Xiangya Hospital, Central South University, Changsha, 410008 China; 6 Department of General Surgery, Xiangya Hospital, Central South University, Changsha, 410008 China; 7 International Joint Research Center of Minimally Invasive Endoscopic Technology Equipment & Standards, Changsha, 410008 China

**Keywords:** Non-small-cell lung cancer, Metastasis

## Abstract

Lung cancer is one of the most lethal malignant tumors in the world. The high recurrence and mortality rate make it urgent for scientists and clinicians to find new targets for better treatment of lung cancer. Early studies indicated that estrogen receptor β (ERβ) might impact the progression of non-small-cell lung cancer (NSCLC). However, the detailed mechanisms, especially its linkage to the CXCR4-mediated cell invasion, remain unclear. Here we found that ERβ could promote NSCLC cell invasion *via* increasing the circular RNA (circRNA), circ-TMX4, expression via directly binding to the 5′ promoter region of its host gene TMX4. ERβ-promoted circ-TMX4 could then sponge and inhibit the micro RNA (miRNA, miR), miR-622, expression, which can then result in increasing the CXCR4 messenger RNA translation via a reduced miRNA binding to its 3′ untranslated region (3′UTR). The preclinical study using an in vivo mouse model with orthotopic xenografts of NSCLC cells confirmed the in vitro data, and the human NSCLC database analysis and tissue staining also confirmed the linkage of ERβ/miR-622/CXCR4 signaling to the NSCLC progression. Together, our findings suggest that ERβ can promote NSCLC cell invasion via altering the ERβ/circ-TMX4/miR-622/CXCR4 signaling, and targeting this newly circ-TMX4/miR-622/CXCR4 signaling may help us find new treatment strategies to better suppress NSCLC progression.

## Introduction

Lung cancer is on the rise globally [1] and is the most frequent cancer and cause of cancer death in men and women combined, and in women, the third most common cancer type and the second most common cause of cancer death [2]. Over the past two decades, a wealth of data has revealed gender differences in the incidence and prognosis of NSCLC. Studies have shown that women who have never smoked are about two and a half times more likely than men to develop lung cancer [3, 4]. Women are more likely to be diagnosed at a younger age than men [5]. There was no significant difference in the incidence of NSCLC between men and postmenopausal women, while the incidence in premenopausal women increased compared with the two groups [5, 6], suggesting that sex hormones (both endogenous and exogenous) play an important role in the occurrence and development of lung cancer [4]. Preclinical studies have found that the expression of estrogen receptors in lung cancer tissues is elevated, suggesting that estrogen is closely related to the incidence of NSCLC [7]. Short-term use of exogenous estrogen is protective, while long-term use increases the risk of lung cancer [8].

Estrogen is a steroidal steroid sex hormone, mainly including estradiol, estriol and estrone, which plays the functional role in promoting sexual organ development, maintaining normal female physiological cycle and promoting bone calcium deposition in the body. Estrogen acts on the corresponding target organs via the estrogen receptor (ER) pathway, exerting the above functions [9]. Estrogen nuclear receptors, including ERα and ERβ, are activated by ligands and translocated into the nucleus from the cytoplasm, binding to the corresponding estrogen response elements, and exert their effects by regulating the transcription of target genes [7]. There are 5 subtypes of ERβ, mainly distributed in ovarian and lung tissue [4]. Studies have shown that ERβ is the main mediating receptor for the physiological effects of estrogen in the lung [10].

Circular RNA (circRNA) is a special type of non-coding RNA molecules. First identified in RNA viruses in the 1970s, circRNAs have long been thought to be results of faulty splicing of mRNAs [11]. In recent years, it has been found that it is a special class of endogenous non-coding RNA molecules, which are usually formed by the back-splicing of some exons and/or introns. CircRNA is ubiquitous in human cells and plays an important role in the regulation of gene expression at the post-transcriptional level [12, 13]. Unlike traditional linear RNA (linear RNA, containing 5′ and 3′ ends), circRNA molecules are in a closed loop structure, which is likely resistant to RNA exonuclease, and has more stable expression and is not easily degraded [14]. CircRNA has been well documented to play important roles in the regulation of tumor initiation, progression, metastasis and chemotherapy resistance [13]. Even though there are some papers published exploring circRNAs’ roles in lung cancer [15, 16], much remains to be done to understand the overall pathophysiological contributions of circRNAs to NSCLC.

As another type of non-coding RNAs, microRNAs (miRNAs) are short in length (20–24 nt), which have been demonstrated to affect the stability and translation of mRNAs through post-transcriptional regulation by binding to the mRNA 3′UTRs of protein-coding genes. MiRNAs have been reported to play important roles in the regulation of the progression of many cancers [13, 17].

CXCR4 is a member of the C-X-C chemokine receptor family, which is associated with multiple types of cancer [18]. CXCR4 plays an important role in lung cancer metastasis, which is a G protein-coupled receptor (GPCR) consisting of 352 amino acids [19], expressed in a variety of cells, selectively binding to its unique ligand CXCL12, also known as stromal cell-derived factor 1 (SDF-1).The binding of CXCL12 to CXCR4 induces intracellular signal transduction through multiple pathways, and plays an important role in cell growth, proliferation, migration and metastasis of breast cancer [20], ovarian cancer [21], colorectal cancer [22] and other tumors.

In this study, we analyzed the changes in the expression of NSCLC-related circRNAs after modulating ERβ levels in NSCLC cells. We identified circ-TMX4 as a downstream target of ERβ that affects NSCLC invasion by acting as a miR-622 sponge and regulating CXCR4 expression, thereby a novel mechanism by which ERβ modulates circ-TMX4/miR-622 signaling to impact NSCLC cell invasion.

## Materials and methods

### Human tissues

Clinical samples of lung cancer and adjacent normal lung tissues were obtained from Department of Thoracic Surgery, Xiangya hospital, Central South University, Changsha, China. All samples were collected for research purpose. The scientific ethics consent was signed by patients before the study.

### Reagents and materials

ERβ, CXCR4, GAPDH antibodies were purchased from Santa Cruz Biotechnology. Anti-mouse/rabbit antibody for Western Blot was from Invitrogen. Normal rabbit IgG was also from Santa Cruz Biotechnology.

### Cell culture and stable cell lines

The human lung cancer cell lines H1299 and A549 were purchased from ATCC, and maintained in RPMI-1640 (Invitrogen) with 10% FBS in the 5% CO_2_ and 37 °C incubator. All cell lines have been tested and authenticated as bacteria and mycoplasma free following ATCC’s instructions during the past 3 months.

### Plasmids construction

The pLKO.1- shcirc-TMX4, pLKO.1-shCXCR4, pWPI-circ-TMX4, pWPI-CXCR4 plasmids, and the psPAX2 packaging plasmid (10ug), and pMD2.G envelope plasmid (10ug), were transfected into 293 T cells using the standard calcium phosphate transfection method. Lentivirus supernatant was collected and concentrated by density gradient after 48 h for immediate use or frozen at −80 °C for later use. 5 µg/ml puromycin was used to select shRNA infected cells. The miR-1303 antisense inhibitor: rArGrArGrCrArArGrArCrCrCrCrGrUrCrUrCrUrArArA, miR-622 antisense inhibitor: rGrCrUrCrCrArArCrCrUrCrArGrCrArGrArCrUrGrU, miR-888 antisense inhibitor: rUrGrArCrUrGrArCrArGrCrUrUrUrUrUrGrArGrUrA. TMX4 mutant promoter construction: F: ATAATCCATCAGTTTTCCCATCTTCTGTTAGAAGTGGATCCACCCACACTC AAGAGGAAAG. R: TGGGAAAACTGATGGATTATCACTTCTTTGTTAGGATCCGAGACTTCC ATTTTGCTGGAA. Mutant circ-TMX4 construction: F: TTACGCCCCATGGTGTCCATCCTGCA CAGGATCCCATTCAGAATGGGAGGCTTTTGC. R: CAGGATGGACACCATGGGGCGTAA. CXCR4 mutant 3’UTR construction: F: TACACATTTTTCAGATATAAAAGGATCCACCAATATT GTACAGTTTTTATT. R: TTTATATCTGAAAAATGTGTA were ordered from the IDT Company.

### Transwell invasion assay

Invasion assay was conducted in 8 µm transwell chamber (Corning Life Science) in 24-well plates. First, 100 µl/well diluted Matrigel (1:20 dilution, BD Biosciences) was coated in the upper chamber with incubation in the 37 °C humidified incubator for 2–4 h. Then, 2–5 × 10^4^ cells/well with serum-free medium was added in the upper chamber and 750 μl media with 10% FBS /well was added into lower chambers. After incubated for 24 h, wash the upper chamber and fix the invaded cells with methanol and stain the cells with 0.1% (w/v) crystal violet. Each sample was conducted in triplicate wells and repeated multiple times.

### Western blot assay

Cells were lysed in RIPA buffer and 30 µg proteins were taken for run on a SDS/PAGE gel. After electrophoresis, the proteins were transferred onto a PVDF membrane (Millipore). After blocking for 1 h and rinsed with TBST for three times, the membrane was incubated in the corresponding primary antibody in a cold room with 4 °C for overnight. Rinse the membrane with TBST for three times again and incubated it with secondary antibodies. Finally, take the images of the membrane by the ECL system (Thermo Fisher Scientific).

### qRT-PCR assay

Trizol reagent (Invitrogen) was used to extract the total RNA of lung cancer cells, and 2 μg of total RNA was used for the reverse transcription. The Bio-Rad CFX96 system was used to conduct and calculate the expression of RNA (mRNA and miRNA). The data were normalized by GAPDH (for mRNA) or U6 (for miRNA) and relative expression was assessed by 2^ΔΔCt^ values. All primers were purchased from Integrated DNA Technologies Company.

### Chromatin immunoprecipitation assay (ChIP)

Normal rabbit IgG (sc-2027, Santa Cruz Biotechnology) and protein A-agarose were used sequentially to preclear the cell lysates. We then added anti-ERβ antibody (2.0 μg) to the cell lysate and incubated overnight at 4 °C. IgG was used in the reaction for the negative control. Specific primer sets were designed to amplify a target sequence within the human TMX4 promoter and agarose gel electrophoresis was used to identify the PCR products.

### Luciferase reporter assay

The human 5′-promoter region of TMX4 was constructed into pGL3-basic luciferase reporter vector (Promega). Deletion mutants without the ERβ binding site in the TMX4 5′ promoter was achieved with the Quick Change mutagenesis. The fragment of CXCR4 3′ UTR with wild-type or mutant miRNA-response elements was cloned into the psiCHECK-2 vector (Promega) downstream of the Renilla luciferase ORF. Cells were plated in 24-well plates and the cDNAs were transfected with Lipofectamine 3000 transfection reagent (Invitrogen, Carlsbad, CA) according to the manufacturer’s instructions. PRL-TK was used as an internal control that served as the baseline control response. Luciferase activity was measured 36–48 h after transfection by Dual-Luciferase Assay (Promega) according to the manufacturer’s manual.

### In vivo studies

1 × 10^6^ cells were suspended in 50 μl serum-free media with 50 μl Matrigel (Becton Dickinson & Co, CA, USA) and injected into the left lateral thorax of the mice as described previously [7, 23]. Tumor development and metastasis were monitored by non-invasive In Vivo Fluorescent Imager (IVIS Spectrum, Caliper Life Sciences) once a week. Mice were sacrificed after 8 weeks, tumors and any metastases were removed for studies. Animal experiments were approved by institutional animal care at Xiangya Hospital Central South University.

### Statistical analysis

All statistical analyses were performed using SPSS 22.0 software. Data were presented as mean ± SD. Differences were analyzed with the one-way ANOVA test, and significance was set at *P* < 0.05.

## Result

### ERβ promotes lung cancer cell invasion through circ-TMX4

**Fig. 1:**
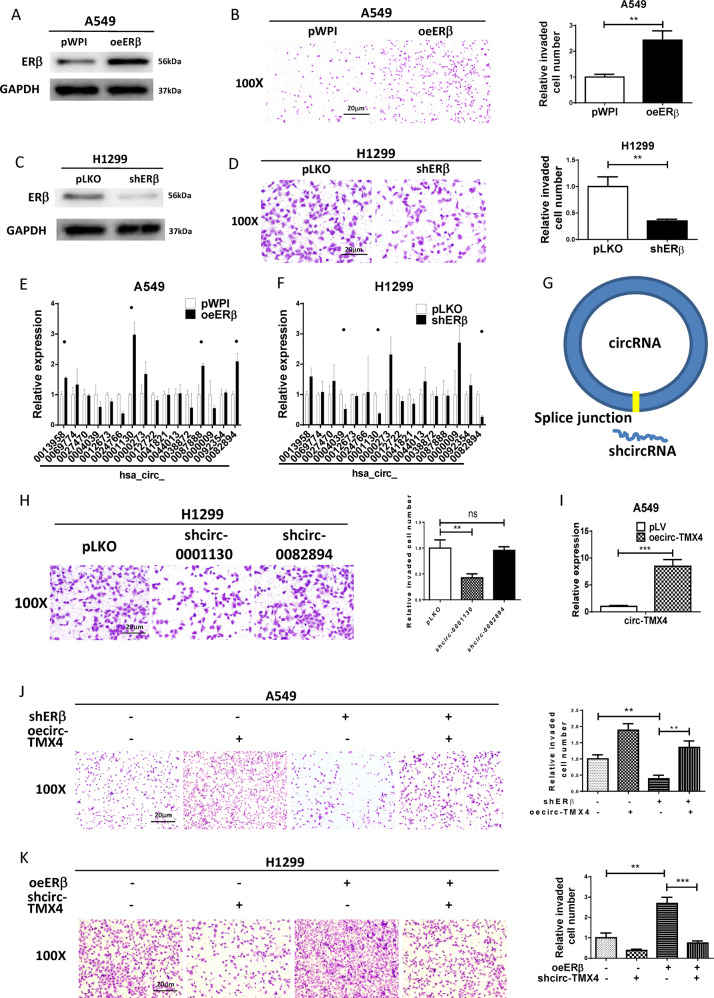
ERβ promotes lung cancer cell invasion though circ-TMX4. **A** Western blot was used to verify ERβ protein expressions after oeERβ in A549. **B** Chamber-transwell invasion assays were performed using A549 cells transfected with oeERβ and pWPI, quantitation is at the right. The invaded cells were counted in five randomly chosen microscopic fields (×100) in each experiment and averaged for quantification. **C** Western blot was used to verify ERβ protein expressions after shERβ in H1299. **D** Chamber-transwell invasion assays were performed using H1299 cells transfected with shERβ and pLKO, quantitation is at the right. **E**, **F** qRT-PCR assay was used to check the top 16 increased circRNAs conducted by high-throughput sequencing in the oeERβ and pWPI(E), shERβ and pLKO(F). **G** Schematic illustration showing the position of the targeting shRNA to knock down the circRNAs (sh-circ) by targeting the specific splice junction. **H** Chamber-transwell invasion assay was used to check the invasion capacity after knocking down circ-0001130 or circ-0082894 in H1299 cells. **I** qRT-PCR assay was used to verify the effect of oecirc-TMX4. **J**, **K** Chamber-transwell invasion assay was used to check the invasion capacity after knocking down ERβ and oecirc-TMX4 in A549(J), oeERβ and shcirc-TMX4 in H1299(K). All quantitations are presented as mean ± SD and *p* values calculated by *t*-test, **p < 0.01, ***p < 0.001, ns = not significant.

While results from recent studies indicated that ERβ can promote lung cancer cell invasion [7], its linkage to circRNA expression for altering lung cancer progression remains unclear. We had applied Western blot assay for ERβ expression in 5 human LCa cell lines (H292, H1299, A549, H358 and H157) (Fig. S1A), and selected H1299 cell line with highest ERβ expression for knock down, and A549 cell line with lower ERβ expression for over expression. We first altered ERβ expression and used Western blot analyses to confirm that ERβ levels were increased after adding ERβ-cDNA (oeERβ) to A549 cells (Fig. 1A), and found that oeERβ promoted A549 cell invasion significantly (Fig. 1B). In contrast, ERβ levels were decreased after adding ERβ-shRNA (shERβ) to H1299 cells with high ERβ expression (Fig. 1C), and shERβ led to decrease significantly lung cancer cell invasion (Fig. 1D).

Using a microarray technique, Zhu X et al. evaluated the circRNA profiles of three paired lung adenocarcinoma (LAC) samples [24]. The results showed up-regulated circRNAs in LCa tissues compared to adjacent normal controls in patients with LAC. In this study, the top 16 circRNAs with significantly increased expression in lung cancer tissues were selected for further analysis. We performed qRT–PCR assays to examine the impact of ERβ on the expression of these 16 circRNAs, and the results revealed that there were 2 circRNAs (hsa_circ_0001130, hsa_circ_0082894) whose expression were consistently altered by increasing or decreasing ERβ levels (Fig. 1E and F).

To further examine whether the 2 circRNAs regulate lung cancer cell invasion, we constructed shRNAs for these 2 circRNAs (sh-circRNA) by targeting specific splice junctions (Fig. 1G). We used qRT–PCR to confirm the knock down efficacy in H1299 (Fig. S1B and C). The results from the transwell assays with matrigel-coated filters revealed that only knocking down hsa_circ_0001130 (circ-TMX4), but not hsa_circ_0082894, could suppress lung cancer cell invasion (Fig. 1H). Furthermore, we also increased the expression of this circRNA and confirmed the efficacy of overexpression in A549 cells (Fig. 1I). We found that over expressing circ-TMX4 could block shERβ-suppressed cell invasion of lung cancer cell (Fig. 1J). Consistent with this, shcirc-TMX4 could also partly reverse ERβ mediated lung cancer cell invasion (Fig. 1K).

Together, the results from Fig. 1A–K suggest that ERβ promotes lung cancer cell invasion through circ-TMX4.

### Circ-TMX4 promotes lung cancer cell invasion

**Fig. 2:**
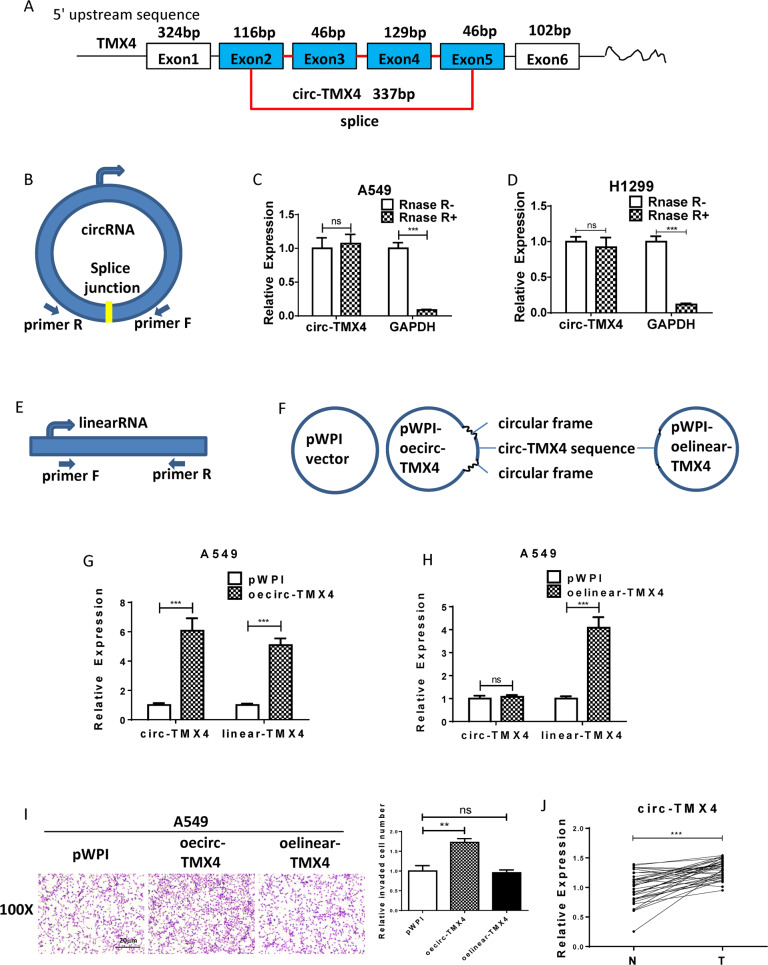
Circ-TMX4 promotes lung cancer cell invasion. **A** The schematic diagram shows the genomic location and splicing patterns of circ-TMX4 (hsa_circ_0001130). **B** The schematic diagram shows the divergent primers covered splice junction to detect circ-TMX4. **C**, **D** The expression of circ-TMX4 and GAPDH mRNA in A549 and H1299 cells treated with or without RNase R was detected by qRT–PCR. **E**, **F** The schematic diagram shows the divergent primers without covered splice junction to detect both linear-TMX4 and circ-TMX4. **G**, **H** qRT-PCR assay was used to verify the effect of oecirc-TMX4 and oelinear-TMX4. **I** Chamber-transwell invasion assay was used to check the invasion capacity after adding circ-TMX4 and linear-TMX4 in A549. **J** The expression of circ-TMX4 in fresh lung cancer tumor tissues (T) and paratumor normal tissues (N) was detected by qRT–PCR. All quantitations are presented as mean ± SD and *p* values calculated by *t*-test, **p < 0.01, ***p < 0.001, ns = not significant.

To further study the impact of ERβ-altered circ-TMX4 on lung cancer cell invasion, we found that circ-TMX4 is derived from exon 2, 3, 4, 5 of TMX4 gene, and has a length of 337 bp (Fig. 2A). To verify that exon2, 3, 4 and 5 of the TMX4 gene formed an endogenous circRNA, we applied qRT–PCR with the divergent primers covering splice junction to detect circ-TMX4 (Fig. 2B), which is resistant to digestion by RNase-R. In contrast, GAPDH mRNA could be significantly decreased after RNase-R digestion (Fig. 2C and D). We also applied qRT–PCR with the divergent primers without covered splice junction to detect both linear-TMX4 and circ-TMX4 (Fig. 2E). In order to demonstrate the specificity of the circ-TMX4 function, we also constructed a linear-formed TMX4 as well as the control vector using the pWPI plasmid (Fig. 2F), and validated their efficacy in A549 cells (Fig. 2G and H). Results from transwell assays confirmed that increasing circ-TMX4 expression increased A549 cell invasion more profoundly than linear-TMX4 sequence compared with the vector control group (Fig. 2I).

To strengthen the above in vitro cell line data, we also studied human clinical lung cancer samples. The results from qRT–PCR assays indicated that circ-TMX4 was significantly elevated in fresh lung cancer tumor tissues (T) compared to the paratumor normal tissues (N) from 36 patients (Fig. 2J).

Together, the results from Fig. 2A–J suggest that circ-TMX4 increases lung cancer cell invasion.

### ERβ regulates circ-TMX4 expression via transcriptional regulation

**Fig. 3:**
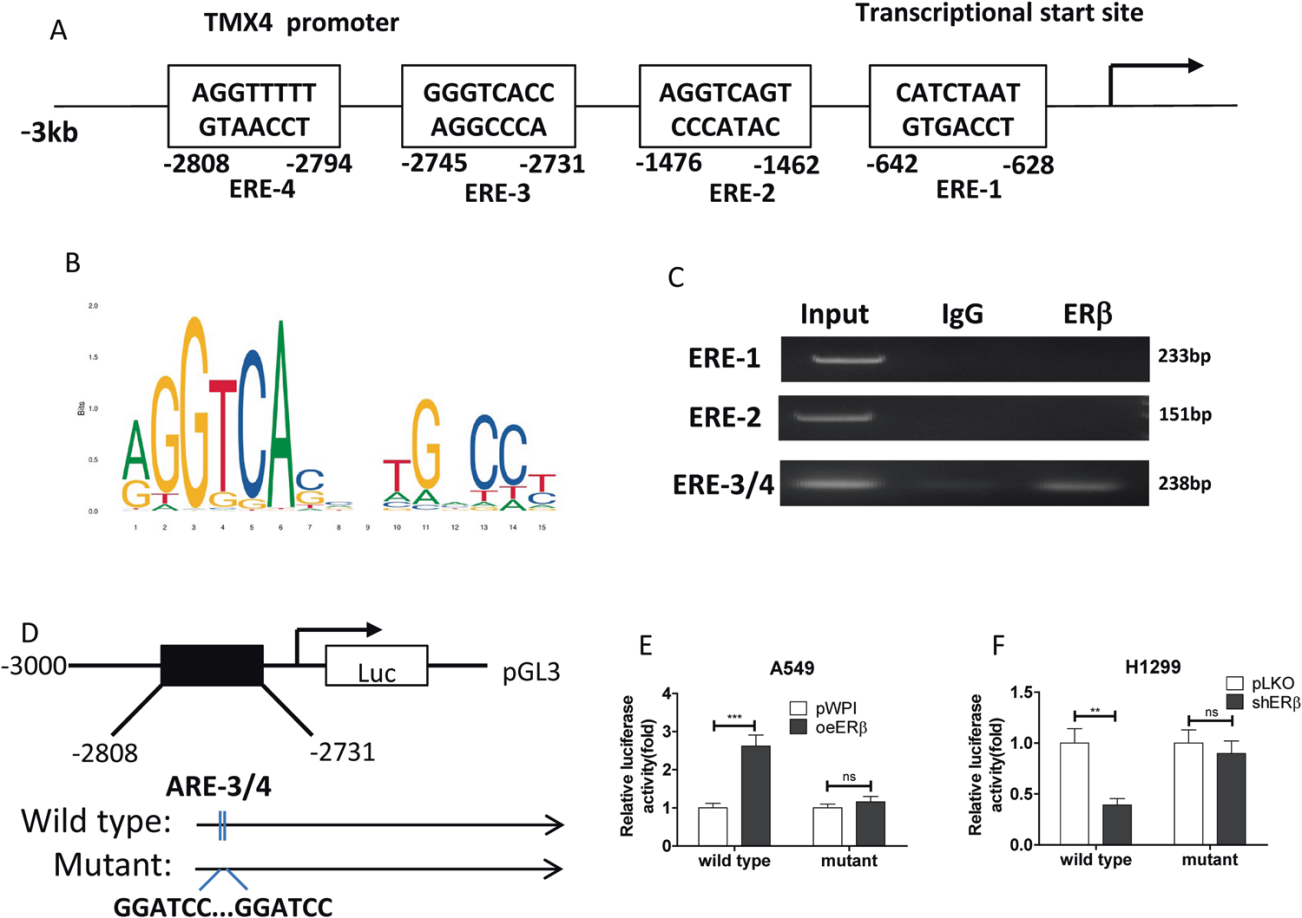
ERβ regulates circ-TMX4 expression via transcriptional regulation. **A**, **B** 4 potential ERβ response elements (EREs) were predicted on the TMX4 3 kb of the 5′-promoter region. **C** Chromatin immunoprecipitation (ChIP) binding assay was performed on H1299. **D** The wild-type and mutant pGL3- TMX4 promoter reporter constructs. **E, F** Luciferase activity after transfection of wild-type or mutant circRNA-SMG1.72 promoter reporter construct in A549 (**E**) cells transfected with oeERβ or pWPI and in H1299 (**F**) cells transfected with shERβ or pLKO. All quantitations are presented as mean ± SD and *p* values calculated by *t*-test, **p < 0.01, ***p < 0.001, ns = not significant.

To dissect the molecular mechanism of how ERβ can regulate the circ-TMX4 expression at the transcriptional level, we relied upon the positive correlation between the host gene (TMX4) expression and its associated circRNA, and applied the Ensembl website (http://asia.ensembl.org/index.html) approaches to search for the potential EREs on the 3 kb region of the host gene TMX4 promoter using JASPAR database, and found 4 putative EREs located within the TMX4 promoter region (Fig. 3A and B). We then applied the ChIP assay to verify their capacity of binding ERβ in H1299 cells, and results revealed that ERβ could bind to the ERE3/4 (Fig. 3C).

Then, to investigate whether ERβ can alter the expression of circ-TMX4 via binding to its host gene promoter, we constructed the TMX4 gene promoter luciferase reporter by inserting a 3 kb 5’ promoter region of TMX4 containing ERE3/4 into the pGL3 luciferase backbone as well as a mutant ERE3/4 (Fig. 3D). As expected, the luciferase assay results revealed that oeERβ significantly increased luciferase activity in A549 cells transfected with wild-type TMX4 promoter, but not in the cells with mutant TMX4 promoter (Fig. 3E). In contrast, shERβ significantly decreased luciferase activity in H1299 cells transfected with wild-type TMX4 promoter, but not in the cells with mutant TMX4 promoter (Fig. 3F).

Together, results from Fig. 3A–F suggested that ERβ could increase circ-TMX4 expression via transcriptional regulation through binding to the ERE3/4 located in its host gene 5′promoter region.

### ERβ/circ-TMX4 signaling can increase lung cancer cell invasion through miR-622

**Fig. 4:**
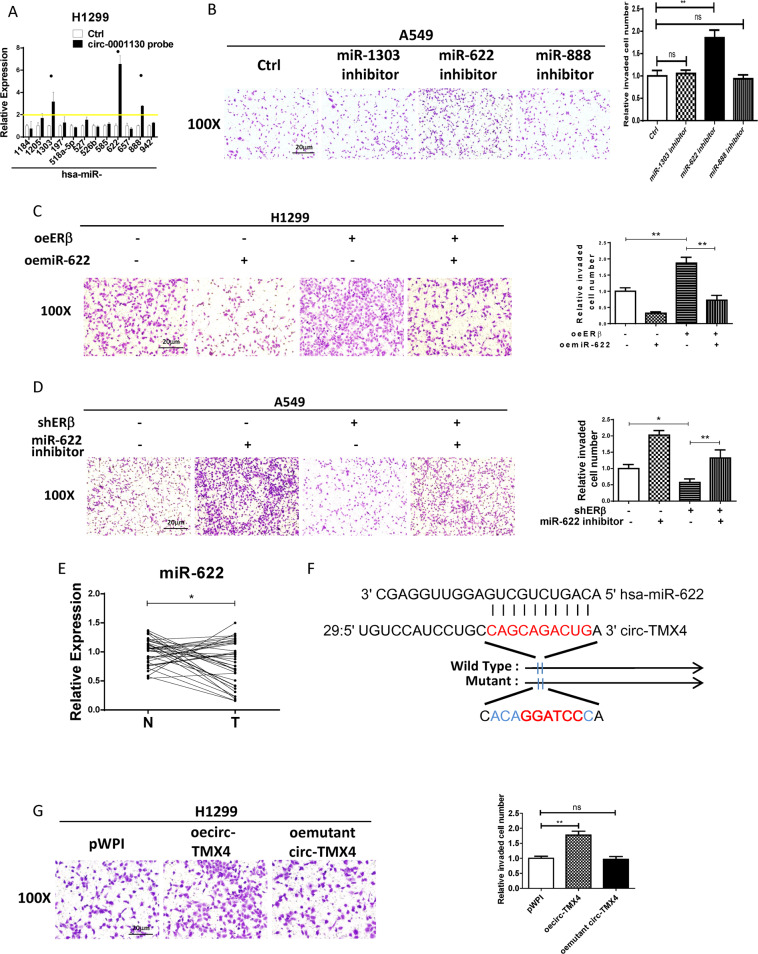
Mechanism dissection of how ERβ/circ-TMX4 signaling can increase lung cancer cell invasion: via altering the miR-622 expression. **A** RNA pull-down assay was applied to test whether circ-TMX4 could interact with 12 potential candidate miRNAs using the biotinylated oligonucleotide to target the circular junction of circ-TMX4. **B** Chamber-transwell invasion assay was used to check the invasion capacity after using miR-1303 inhibitor, miR-622 inhibitor and miR-888 inhibitor. **C**, **D** Chamber-transwell invasion assay was used to check the invasion capacity in H1299 (**C**) cells transfected with oeERβ or oemiR-622 and in A549 (**D**) cells transfected with shERβ or miR-622 inhibitor. **E** The expression of miR-622 in fresh lung cancer tumor tissues (T) and paratumor normal tissues (N) was detected by qRT–PCR. **F** The wild-type and mutant oecirc-TMX4 constructs. **G** Chamber-transwell invasion assays showed the efficiency of wild-type and mutant oecirc-TMX4 in H1299 cells. All quantitations are presented as mean ± SD and *p* values calculated by *t*-test, *p < 0.05, **p < 0.01, ns = not significant.

As circRNAs may act as competing endogenous RNA (ceRNA) to regulate other RNA transcripts by competing for shared miRNAs [25], using analysis from databases (Circular RNA Interactome), we were able to identify top 12 potential candidate miRNAs with high scores. We then applied the RNA pull-down assay to test whether circ-TMX4 could interact with these candidate miRNAs using the biotinylated oligonucleotide (5′-CCATGGGGCGTAACCATATC-3′) to target the circular junction of circ-TMX4. Results revealed that 3 miRs (miR-1303, miR-622, miR-888) were enriched in the pull-down assay, suggesting the potential direct binding of the 3 miRs with circ-TMX4 (Fig. 4A). Yet, only miR-622 inhibitor could promote lung cancer cell invasion (Fig. 4B), therefore we focused on the functional interaction between circ-TMX4 and miR-622. The results from the rescue assay in H1299 cells revealed that the oeERβ-increased lung cancer cell invasion could be partially reversed via oemiR-622 (Fig. 4C), and shERβ-decreased lung cancer cell invasion could be partially blocked via treating the A549 cells with the miR-622 inhibitor (antisense construct) (Fig. 4D). The results from the rescue assay in H1299 cells revealed that the oecirc-TMX4-increased lung cancer cell invasion could be partially reversed via oemiR-622 (Fig. S1D).

We also examined the level of miRNA-622 in human clinical lung cancer samples. The results from qRT–PCR assays indicated that miR-622 was significantly decreased in fresh lung cancer tumor tissues (T) compared to paratumor normal tissues (N) from 36 patients (Fig. 4E).

To dissect the mechanism of how circ-TMX4 can act on miR-622, we identified the miR-622 binding sites located on the circ-TMX4 (Circular RNA Interactome), and then constructed a mutant circ-TMX4 (Fig. 4F) without the miRNA binding site. As expected, addition of mutant circ-TMX4 in H1299 cells failed to increase significantly lung cancer cell invasion (Fig. 4G), suggesting that circ-TMX4 promoted lung cancer cell invasion via sponging and inhibition of miR-622.

We also collected the clinicopathological data of lung cancer patients who underwent surgery in our hospital in the past two years (2019-2020), and the analysis showed that tumor size, T grade, N grade and differentiation had significant correlations with circ-TMX4 and/or miR-622 expression (Table 1).

**Table 1:** Correlation between circ-TMX4/miR-622 expression and clinical pathologic characteristics.

Characteristics	Case	circ-TMX4		*p*	miR-622		*p*
		low	high		low	high	
All cases	128	64	64		64	64	
Age (years)							
<60	78	38	40	0.717	41	37	0.469
≥60	50	26	24		23	27	
Gender							
Male	75	37	38	0.858	39	36	0.590
Female	53	27	26		25	28	
Smoking							
No	43	20	23	0.575	24	19	0.349
Yes	85	44	41		40	45	
Tumor size (cm)							
<3	76	48	28	<0.001^*^	34	42	0.150
≥3	52	16	36		30	22	
T grade							
T1–T2	85	55	30	<0.001*	33	52	<0.001*
T3–T4	43	9	34		31	12	
N grade							
N0-N1	87	58	29	<0.001*	32	55	<0.001*
N2-N3	41	6	35		32	9	
Differentiation							
Well	80	45	35	0.068	28	52	<0.001*
Moderate-Poor	48	19	29		36	12	

^*^Significant association.

Bold values indicates statistically significant *p* < 0.05 values.

Together, results from Fig. 4A–G and Table 1 suggest ERβ/circ-TMX4 axis may function via interacting/ altering miR-622 to impact the lung cancer cell invasion.

### ERβ/circ-TMX4/miR-622 axis promoted lung cancer cell invasion *via* altering CXCR4 expression

**Fig. 5:**
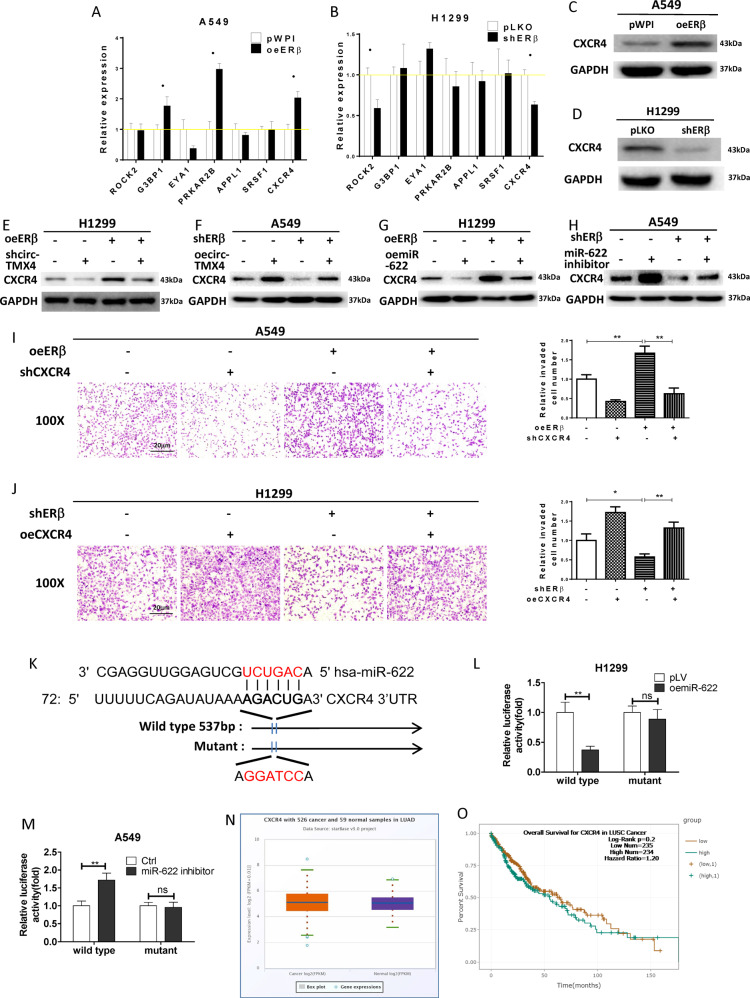
ERβ/circ-TMX4/miR−622 axis promoted lung cancer cell invasion via altering CXCR4 expression. **A**, **B** The qRT-PCR assay for screening metastasis-associated genes in A549 cells with oeERβ compared with pWPI (**A**) and in H1299 cells with shERβ comparing with vector pLKO (**B**). **C**, **D** Western blot assay for CXCR4 expression in A549 cells with oeERβ or control (**C**) and in H1299 cells with knocked down ERβ or control (**D**). **E**–**H** Western blot assays were performed to detect CXCR4 expression in 2 cell lines by manipulating ERβ/circ-TMX4 and ERβ/miR-622. **I**, **J** Chamber-transwell invasion assay was used to check the invasion capacity in A549 (**I**) cells transfected with oeERβ or shCXCR4 and in H1299 (**J**) cells transfected with shERβ or oeCXCR4. **K** Sequence alignment of the CXCR4 3′UTR with wild-type versus mutant without miR-622 targeting sites. **L**, **M** Luciferase reporter activity after transfection of wild-type or mutant CXCR4 3′UTR reporter construct in H1299 cells with/without oemiR-622 (**L**), and in A549 cells treated with/without miR-622 inhibitor (**M**). **N** Lung cancer patients’ data from TCGA show different expression of CXCR4 in normal (**N**) tissues and tumor (T) tissues. **O** Lung cancer patients’ data from TCGA show the relationship between expression of CXCR4 and overall survival (OS) rates. All quantitations are presented as mean ± SD and *p* values calculated by *t*-test, *p < 0.05, **p < 0.01, ns = not significant.

To further dissect how the ERβ/circ-TMX4/miR-622 axis can enhance lung cancer cell invasion, we searched for metastasis genes that are linked to circ-TMX4 or miR-622 in the lung cancer cell databases (circBase, CircNet and OncoLnc). In brief, we used these websites to predict the candidate metastasis genes which may regulate lung cancer invasion. Based on different algorithms and criteria, each website could predict a panel of candidate metastasis genes with different matching scores. Among those panels of metastasis genes, we select 7 candidates which could be identified by 2 or more websites and with higher matching scores. We focused on the 7 metastasis-related genes, ROCK2, G3BP1, EYA1, PRKAR2B, APPL1, SRSF1 and CXCR4 to further test their expressions with qRT-PCR. Comparing the differential expressions of the genes in the 2 cell lines, the results revealed that CXCR4 had the differential change with oeERβ in A549 cells increasing the expression of G3BP1, PRKAR2B, CXCR4 (Fig. 5A) and shERβ in H1299 cells decreasing the expression of ROCK2 and CXCR4 (Fig. 5B), and western blot and invasion data also confirmed this relationship in 3 cell lines (Figs. 5C and D, S1E−L), so we decided to focus on the CXCR4 to further study its linkage to the ERβ, circ-TMX4 and miR-622.

We confirmed that knocking down circ-TMX4 decreased the CXCR4 protein expression and could partly reverse the oeERβ-promoted CXCR4 expression in H1299 cells (Fig. 5E), whereas in A549 cells, adding circ-TMX4 increased the CXCR4 protein expression and could effectively reverse the shERβ-decreased CXCR4 expression (Fig. 5F). Importantly, we found that adding miR-622 in H1299 cells decreased the CXCR4 expression and effectively reversed the ERβ-increased CXCR4 expression (Fig. 5G), while treating A549 cells with miR-622 inhibitor increased CXCR4 expression and partly reversed the ERβ-shRNA-suppressed CXCR4 expression (Fig. 5H). We also treated H1299 cells with oecirc-TMX4 and AMD3100 (CXCR4 Antagonist), A549 cell with shcirc-TMX4 and CXCL12 (SDF-1α, CXCR4 Ligands), then detected functional CXCR4 expression by Fluorescence activated Cell Sorting (FACS). We found that AMD3100 decreased the functional CXCR4 expression and could reverse the oecirc-TMX4-promoted CXCR4 expression in H1299 cells (Fig. S2A), whereas in A549 cells, CXCL12 increased the functional CXCR4 expression and could effectively reverse the shcirc-TMX4-decreased CXCR4 expression (Fig. S2B).

Next, to examine the consequences after suppressing the CXCR4 expression in lung cancer cells, we applied the interruption approach using CXCR4-shRNA. The rescue experiments *via* Chamber-transwell invasion assay results revealed that CXCR4 knockdown by shRNA could effectively reduce the ERβ-enhanced lung cancer cell invasion in A549 cells (Fig. 5I), and shERβ-decreased lung cancer cell invasion could be partially blocked via treating the H1299 cells with oeCXCR4 (Fig. 5J).

For mechanism dissection of how miR-622 can modulate CXCR4 expression at the molecular level, we identified the potential binding site located on the 3’UTR of CXCR4-mRNA (http://www.targetscan.org/vert_71/). We then applied the reporter assay with the psiCHECK2 vector carrying the wild-type (WT) miRNA-target sites and mutant without miRNA-target sites (Fig. 5K), and results revealed that miR-622 could decrease the luciferase reporter activity of wild-type 3’UTR of CXCR4, with little effect on the mutant 3’UTR of CXCR4-mRNA in H1299 cells (Fig. 5L). In contrast, miR-622 inhibitor significantly increased luciferase activity in A549 cells transfected with wild-type 3’UTR of CXCR4, but not in the cells with mutant 3’UTR of CXCR4-mRNA (Fig. 5M), suggesting that miR-622 could directly target the 3’UTR of CXCR4-mRNA to suppress the protein expression.

Based on TCGA database, the data from human clinical sample surveys showed a general trend that CXCR4 was increased in lung cancer tumor tissues compared to paratumor normal tissues (Fig. 5N) while patients with lower CXCR4 expression tended to have better overall survival in the first 100 months after diagnosis of lung cancer (Fig. 5O), although a statistical significance was not reached (*p* = 0.2).

Together, results from Fig. 5A–O suggest that ERβ/circ-TMX4/miR-622 axis enhanced lung cancer cell invasion *via* altering the CXCR4 expression.

### Preclinical study using the in vivo mouse model to demonstrate the role of ERβ/circ-TMX4/miR-622/CXCR4 signaling in the lung cancer progression

**Fig. 6:**
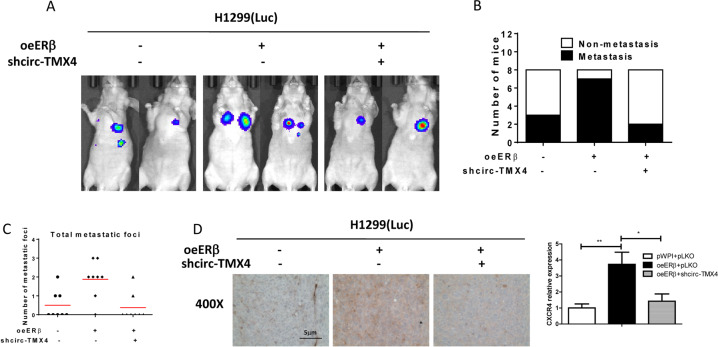
Preclinical study using the in vivo mouse model to demonstrate the role of ERβ/circ-TMX4/miR−622/CXCR4 signaling in the lung cancer progression. **A** IVIS imaging was used to detect the various distal metastasis foci in mice. **B** Quantification of the mice with metastasis. **C** Quantification of the total metastatic foci. **D** IHC assay was used to check CXCR4 expression in mice tumor tissues in each group. All quantitations are presented as mean ± SD and *p* values calculated by *t*-test, *p < 0.05, **p < 0.01.

To test whether the mechanisms that we uncovered in vitro similarly play a significant role in vivo, we injected 1×10^6^ H1299-Luc cells into the left lateral thorax of nude mice in situ in the xenografted mouse model. The mice were divided into three groups: 1)pWPI+pLKO; 2)oe ERβ + pLKO; 3)oeERβ + shcirc-TMX4. Tumor development and metastasis were monitored by non-invasive In Vivo Fluorescent Imager (IVIS Spectrum, Caliper Life Sciences) once a week. Mice were sacrificed after 8 weeks, tumors and any metastases were removed for studies. The results showed that the addition of ERβ could promote lung cancer metastasis, and shcirc-TMX4 could block ERβ mediated progression of lung cancer (Fig. 6A). We then sacrificed the mice and counted the number of metastatic foci, and the results also consistent with the IVIS signal (Fig. 6B and C). We have determined the expression of miR-622 in in vivo xenograft tumors harvested from mice. The results showed that neither oeERβ nor shcirc-TMX4 can change miR-622 expression significantly (Fig. S2C). IHC data also confirmed that shcirc-TMX4 could also reduce CXCR4 expression mediated by ERβ (Fig. 6D). We also confirmed the signaling in A549 -Luc cells with 3 groups: (1) pWPI+Ctrl; (2) oeERβ + Ctrl; (3) oeERβ + oemiR-622. We treated the mice as above, and the result showed that oemiR-622 could block ERβ mediated progression of lung cancer (Fig. S2D–F).

Together, the results from our preclinical study using in vivo mouse model in Fig. 6A–D prove that ERβ may play an important role to promote the liver cancer metastasis via altering the circ-TMX4/CXCR4 signaling axis.

## Discussion

In the present study, we demonstrated that ERβ could promote NSCLC cell invasion via increasing the circular RNA (circRNA), circ-TMX4, expression via direct binding to the 5’ promoter region of its host gene TMX4. ERβ-promoted circ-TMX4 could then sponge miR-622 and inhibit its function, which can then result in increasing the CXCR4 messenger RNA translation via a reduced miR binding to its 3’UTR.

There have been several reports on the roles of ERβ in NSCLC progression [7, 26–29]. While the majority of clinical evidence pointed to a tumor-promoting role of ERβ [7, 26, 27], ERβ was also reported to have a protecting effect for cancer progression [30]. A study found that hormone replacement therapy had a strong negative effect on survival after diagnosis of lung cancer [31]. How ERβ affects the progression of NSCLC remains controversial [32–34]. Some research revealed ERβ expression is a cancer-promoting gene while others not. On the other hand, estrogen has been proved to be important factors in promoting the progression of NSCLC through enhancing epithelial-mesenchymal transition (EMT) [35].

In our study, we found that circRNAs are important regulatory molecules in lung cancer progression. ERβ could promote NSCLC cell invasion *via* increasing circ-TMX4 expression because ERβ could directly bind to the 5’ promoter region of its host gene TMX4. A large number of studies have found that non-coding RNA (ncRNA) plays an important role in the occurrence and development of NSCLC [7, 36, 37], of which circular RNA (circRNA) is one of the current active research areas. More and more studies have found that circRNAs can regulate different functions through interaction with microRNAs (miRNAs) [38–40]. Sang et al. reported that circRNA_0025202 could regulate breast cancer tamoxifen sensitivity and tumor progression via regulating the miR-182-5p/FOXO3a axis [41]. Zeng et al. showed that CircHIPK3 could promote colorectal cancer growth and metastasis by sponging miR-7 [42]. Our study has shown that circ-TMX4 could suppress the function of miR-622 via sponging it but not changing its expression level.

MiRNAs plays an important role in the development of tumors [13, 43–46]. miRNAs have been shown to negatively mediate the expression of their target genes by inducing mRNA degradation or inhibiting protein translation mainly through binding with 3’UTR [47, 48]. Our result showed that miR-622 can decrease the CXCR4 messenger RNA translation via a reduced miRNA binding to its 3’UTR. Conversely overexpressing miR-622 or adding miR-622 inhibitor also confirmed the validity of this signaling. In our study, the data showed the reverse function was partial, suggesting that sponging miR-622 is only one of the signaling pathway engaged by ERβ-circ-TMX4 for lung cancer progression, others mechanisms remain to be determined.

Our analysis of the online TCGA database on NSCLC samples shows significant tumor-promoting effects of CXCR4. Many NSCLC cell lines express high levels of CXCR4, and SDF-1-activated CXCR4 promotes in vitro migration and invasion of these cell lines [49]. In vivo experiments confirmed that blocking the CXCR4/SDF-1 pathway can inhibit NSCLC cell metastasis [50]. Some retrospective studies have reported the relationship between the expression of CXCR4 in NSCLC and clinical prognosis [51, 52], suggesting that the CXCR4/SDF-1 pathway plays an important role in NSCLC. Since NSCLC is a group of malignant tumors with wide heterogeneity, the prognosis may be different according to different subtypes. Indeed it was found that CXCR4 is highly expressed in many specimens of lung adenosquamous carcinoma. In addition, the high expression of CXCR4 is often associated with tumor metastasis and poor prognosis [53]. Our study also proved that CXCR4 was an oncogene under the regulation of ERβ/circ-TMX4/miR-622 signaling to promote lung cancer progression, thus our work established the foundation to target this signaling for developing new drugs for the better treatment of lung cancer.

## Supplementary information

Supplementary Figure legend

Figure S1

Figure S2

Reproducibility checklist

## Data Availability

The data used to support the findings of this study are included within the article.
