# Supplementary material for: Estrogen receptor beta promotes lung cancer invasion via increasing CXCR4 expression
Source: Cell Death Dis. 2022 Jan 21;13(1):70. doi: 10.1038/s41419-022-04514-4 (PMC8782891; doi:10.1038/s41419-022-04514-4)
Supplement: Supplementary file 1 — Supplementary Figure legend [file 41419_2022_4514_MOESM1_ESM.docx]

**Supplementary figure legend**

**Figure S1**

**A.** Western blot was used to detect ERβ protein expression in 5 human LCa cell lines (H292, H1299, A549, H358 and H157). **B-C.** qRT-PCR assay was used to check circ-TMX4, linear-TMX4 and circ-0082894 level after shcircRNA in H1299. **D.** Chamber-transwell invasion assay was used to check the invasion capacity in H1299 cells transfected with oecirc-TMX4 or oemiR-622. **E.** Western blot was used to detect CXCR4 protein expression after oeERβ in H1299. **F.** Chamber-transwell invasion assays were performed using H1299 cells transfected with oeERβ and pWPI, quantitation is at the right. **G.** Western blot was used to detect CXCR4 protein expression after oeERβ in H358. **H.** Chamber-transwell invasion assays were performed using H358 cells transfected with oeERβ and pWPI, quantitation is at the right. **I.** Western blot was used to detect CXCR4 protein expression after shERβ in A549. **J.** Chamber-transwell invasion assays were performed using A549 cells transfected with shERβ and pLKO, quantitation is at the right. **K.** Western blot was used to detect CXCR4 protein expression after shERβ in H358. **L.** Chamber-transwell invasion assays were performed using H358 cells transfected with shERβ and pLKO, quantitation is at the right. All quantitations are presented as mean ± SD and p values calculated by t-test, *p < 0.05, **p < 0.01, ns = not significant.

.

**Figure S2**

**A.** FACS was used to detect functional CXCR4 protein expression after treating with oecirc-TMX4 and AMD3100 in H1299. **B.** FACS was used to detect functional CXCR4 protein expression after treating with shcirc-TMX4 and CXCl12 in A549. **C.** qRT-PCR assay was used to determine the expression of miR-622 in in vivo xenograft tumors harvested from mice. **D.** IVIS imaging was used to detect the various distal metastasis foci in mice. **E.** Quantification of the mice with metastasis. **F.** Quantification of the total metastatic foci. All quantitations are presented as mean ± SD and p values calculated by t-test, *p < 0.05, ns = not significant.
