# Supplementary figures and images for: Estrogen receptor beta promotes lung cancer invasion via increasing CXCR4 expression
Source: Cell Death Dis. 2022 Jan 21;13(1):70. doi: 10.1038/s41419-022-04514-4 (PMC8782891; doi:10.1038/s41419-022-04514-4)

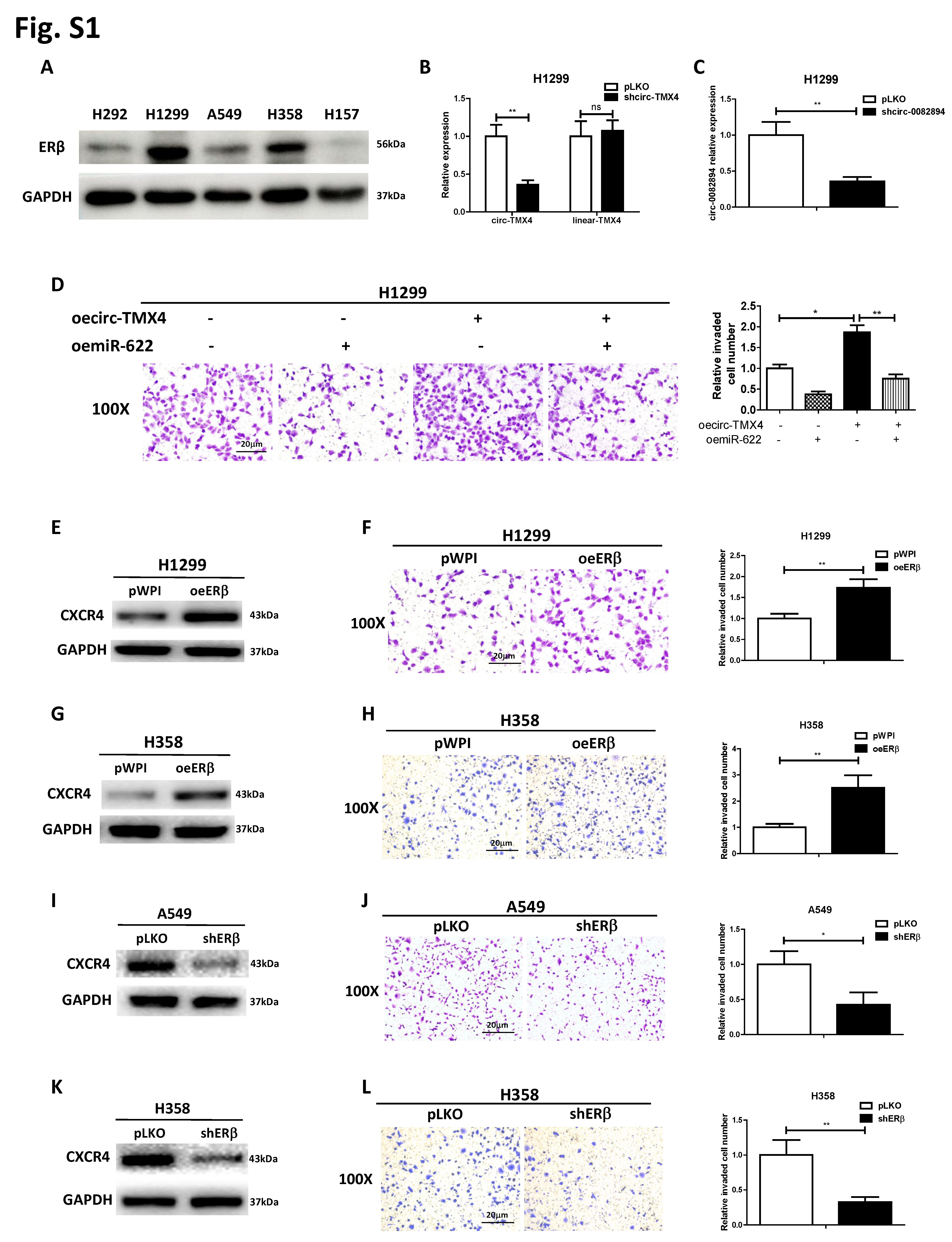

Supplement: Supplementary file 2 — Figure S1 [file 41419_2022_4514_MOESM2_ESM.tif]
